# Overview of global healthcare policies for patients with chronic kidney disease: an integrative literature review

**DOI:** 10.31744/einstein_journal/2024RW0519

**Published:** 2024-07-02

**Authors:** Letícia Borges Mendonça Soares, Alcimar Barbosa Soares, Janise Braga Barros Ferreira

**Affiliations:** 1 Postgraduate Program in Public Health Faculdade de Medicina de Ribeirão Preto Universidade de São Paulo Ribeirão Preto SP Brazil Postgraduate Program in Public Health, Faculdade de Medicina de Ribeirão Preto, Universidade de São Paulo,Ribeirão Preto, SP, Brazil.; 2 Program in Biomedic Postgraduate al Engineering Faculdade de Engenharia Elétrica Universidade Federal de Uberlândia Uberlândia MG Brazil Program in Biomedic Postgraduate al Engineering, Faculdade de Engenharia Elétrica, Universidade Federal de Uberlândia, Uberlândia, MG, Brazil.

**Keywords:** Kidney failure, chronic, Renal insufficiency, chronic, Public health, Public policy, Universal health care, Health services accessibility

## Abstract

**Introduction:**

Chronic kidney disease is a progressive and irreversible loss of kidney function and considerably affects the lives of patients and their families. Its high incidence necessitates efficient public policies for prevention and treatment. However, policies for chronic kidney disease education and awareness are scarce.

**Objective:**

To evaluate global public policies for the prevention and treatment of chronic kidney disease adopted in various regions, aiming to comprehend the differences between various models.

**Methods:**

This integrative review followed PRISMA recommendations and included papers published between 2016 and 2021 across several databases.

**Results:**

The 44 selected articles were categorized into three themes: structural and financial aspects of the organization of renal healthcare, access to renal healthcare or management of chronic kidney disease, and coping strategies for chronic kidney disease or kidney health. Critical analysis of the papers revealed global neglect of kidney disease in political agendas. Considerable policy variations exist between different countries and regions of the same country. Our research highlighted that free and universal health coverage, especially for the most vulnerable patients, is crucial for accessing treatment owing to the prohibitively high treatment costs.

**Conclusion:**

Social, economic, and ethnic inequalities strongly correlate with disease occurrence, primarily affecting minority groups who lack health support, especially for the prevention and treatment of chronic kidney disease.

## INTRODUCTION

Current estimates indicate a global prevalence of chronic kidney disease (CKD) in stages 1 to 5 at 14.3% in the general population and 36.1% in high-risk groups.^(1,2)^ Despite such alarming numbers, global public policies for preventing kidney diseases are relatively recent. The first guidelines for the diagnosis and treatment of CKD were published in 2002 by the National Kidney Foundation in a document entitled the Kidney Disease Outcomes Quality Initiative.^(3)^ Recently, the Global Kidney Health Atlas (a study conducted in 118 countries) identified considerable shortcomings in renal care in most countries, especially at the primary care level.^(4)^ Among low-income countries, particularly in Africa, only a third are capable of providing basic assessment tests such as serum creatinine measurement, with none equipped to measure albuminuria and the estimated glomerular filtration rate (eGFR), crucial for the diagnosis and staging of CKD.^(4,5)^ This is also present in high-income countries, where only 58% and 68% of patients in primary care can provide information on albuminuria and eGFR, respectively.^4^ Notably, the treatment of CKD, especially in stages 3 to 5, is costly and inaccessible for much of the global population without the support of public policies and programs.^(6-8)^ For example, 79% of patients undergoing dialysis are funded by the Public Health System (SUS - *Sistema Único de Saúde*), as per the 2019 Brazilian Dialysis Survey.^(9)^ The survey also indicated increasing incidence and prevalence of patients undergoing dialysis. However, notable inequities exist between the states and regions of the country, suggesting major limitations in treatment access.^(9)^

Backman et al. noted that among the 194 countries studied, only 56 have constitutional provisions for citizens’ right to health,with many needing to improve the delivery of these rights stated in their Constitutions.^(10)^ According to the World Health Organization (WHO), access to essential health services has improved over the last decade. Nevertheless, coverage in low- and middle-income countries remains well below the average for wealthier countries. In 2017, only 33%-49% of the world’s population could access essential health services.^(7,11)^

A rapid increase in mortality is associated with non-communicable diseases (NCDs).^(1,6,7,11,12)^ In 2016, 71% of global deaths were atttributed to NCDs, with 85% of premature deaths occurring among people aged 30-70 years in low- and middle-income countries.^(11,13)^Despite its high treatment costs and significant mortality rates, CKD is not positioned as a top-priority NCD by international organizations, despite affecting over 750 million adults annually.^(14,15)^ For example, recent reports published by the WHO in 2019 and 2020 notably omit CKD, unlike other NCDs such as cardiovascular diseases, cancer, chronic respiratory diseases, and diabetes.^(11,13)^ This discrepancy is evident in discussions within other world associations, such as the International Society of Nephrology, which declared CKD as one of the leading global health challenges.^(5,16,17)^This raises questions about whether the limited emphasis on CKD by prominent international organizations such as the WHO also affects the public policies of various countries regarding CKD.^(17)^ Our hypothesis is also supported by several studies reporting that although many countries have national policies and strategies for coping with NCDs, these tend to vary considerably depending on the type of disease. Specific policies aimed at education and awareness regarding the importance of screening, managing, and treating CKD are rare.^(5,12,13,16-18)^

## OBJECTIVE

Therefore, this study aimed to evaluate the public policies for the prevention and treatment of chronic kidney disease adopted by different nations, seeking to understand the differences among the models implemented worldwide.

## METHODS

### Research strategy and screening of articles

An integrative literature review was performed by iterating the following phases: a) identification of the theme and definition of the guiding question; b) literature search and selection strategy; c) categorization, evaluation, and analysis of the articles; and d) writing of the review paper.

The bibliographic survey focused on 2016-2021 to address the central research question effectively. The search, conducted from January to April 2021, aimed to answer: “What are the strategic guidelines of public policies for addressing chronic kidney disease (CKD) in various global regions?”

Analysis of the collected material was conducted between May and June 2021. The following databases were explored: PubMed^®^, EMBASE^®^ (Excerpta Medica Database), and Scopus^®^ (SciVerse Scopus).

### Strategy

Searches were directed by controlled descriptors, using terms such as ‘Chronic Kidney Disease’, ‘Chronic Kidney Failure,’ ‘Chronic Renal Failure,’ ‘Chronic Renal Disease,’ ‘Public Health,’ ‘Policy,’ and ‘System,’ combined with boolean operators AND and OR across selected databases.

### Selection criteria

The key inclusion criteria were as follows: i) academic papers; ii) published in journals with an abstract and full text; iii) available in Portuguese, English, or Spanish; and iv) adopted an empirical method of investigation of the topic under analysis. The exclusion criteria were as follows: i) studies addressing only non-adult populations; ii) duplicate articles; and iii) studies not directly related to the central theme of this review.

### Data extraction and analysis

Study data was extracted into a Microsoft Excel spreadsheet using EndNote software and categorized as follows: author, year of publication, country, title, journal, method, conclusion, and study focus. The papers were subsequently grouped by thematic categories to analyze and compare how CKD is addressed globally.

## RESULTS

### Research and article section process

**Figure 1:**
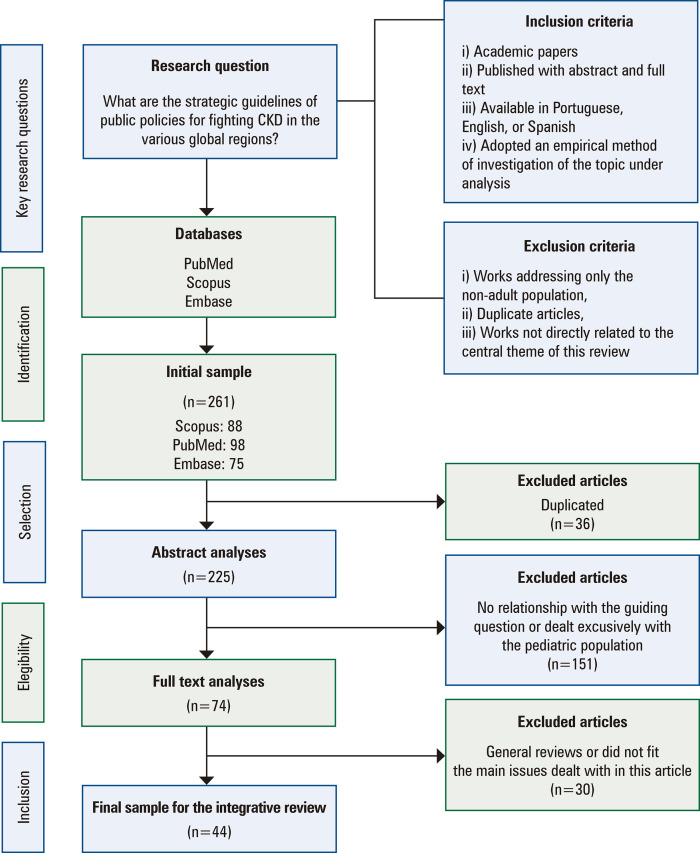
Flowchart of the paper selection strategy for this integrative review

Figure 1 presents the steps of the integrative review and article selection strategies.

CKD: chronic kidney disease.

The search yielded 261 articles. The initial scan identified 36 duplicate items, which were removed. The abstracts of the remaining 225 publications were evaluated, excluding 151 articles that showed no relationship with the guiding question or focused on non-adult populations. The remaining 81 articles were read in full, leading to the exclusion of 30 papers that presented general reviews or focused on irrelevant aspects. To minimize possible biases, selection was performed by two authors. Impasses regarding inclusion or exclusion of articles were resolved through discussion, consensus, or consultation with a third researcher.

Consequently, 44 articles derived from descriptive and/or qualitative studies were included in this review.

### Characteristics of included studies

**Figure 2:**
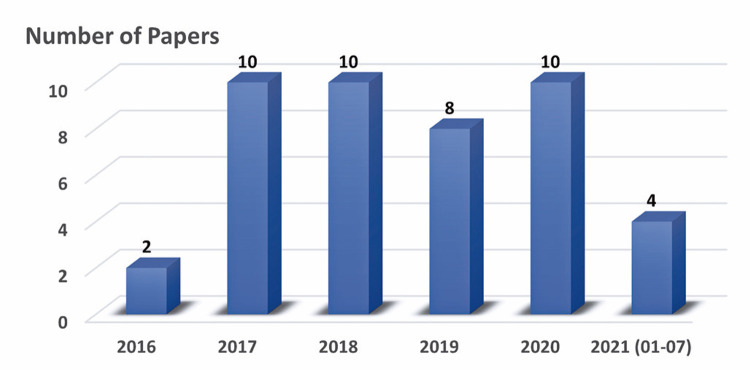
Number of papers included in this review, distributed over the years

**Figure 3:**
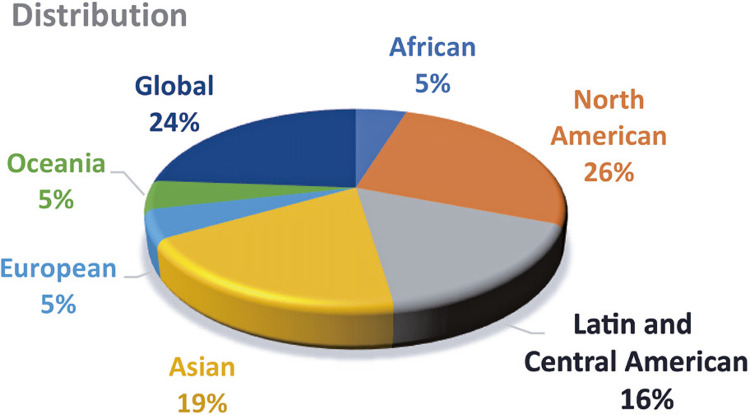
Geographic distribution of selected papers

The main characteristics of the studies selected for this review are highlighted in figures 2 and 3, and detailed in table 1A to C [Table t2] [Table t3].

**Table 1A:** Papers classified into the main thematic category “Structural and financial aspects associated with how renal healthcare is managed at the organizational level”

Theme	Authors	Objectives	Main findings
Public expenditure and budget for renal healthcare	Goncalves et al.(2018)^(19)^	To estimate the cost of chronic kidney disease (CKD) and end-stage renal disease (ESRD) attributed to diabetes in Brazil, stratified by sex, race, skin color, and age.	Diabetes was responsible for 22% of the total cost related to CKD and ESRD; The largest share of costs attributable to CKD was hemodialysis (HD) and peritoneal dialysis (PD). The economic burden of CKD may increase in future, posing severe implications for the financial sustainability of the Brazilian public health system.
	Ismail et al. (2019)^(20)^	To quantify the economic burden of ESRD on the Malaysian healthcare system.	The total annual public expenditure on ESRD grew by 94% over a 7-year period; In 2010, spending on ESRD constituted 2.95% of total public sector health expenditure. In 2016, the proportion increased to 4.2%; with 6% of ESRD expenditures allocated to kidney transplantation and 94% to dialysis.
	Afiatin et al. (2017)^(21)^	To assess the cost-effectiveness and budgetary impact of the HD-first policy compared to the PD-first in Indonesia.	The budget required for the PD first policy was 50% less than that for the HD first policy.
	Ismail et al. (2020)^(22)^	To compare the economic burden of ESRD on the national health systems in countries with high prevalence according to the US Renal Data System (USRDS), including Malaysia.	Expenditure on ESRD accounts for 0.91% to 7.1% of the total national health system expenditures in high-prevalence countries; In Malaysia, the public sector paid for 70% of dialyses; Expenditure on ESRD in Malaysia accounts for 4.2% of the total public health expenditure.
	van der Tol et al. (2019)^(23)^	To compare global government reimbursements for dialysis costs.	90% of the responding countries (n = 90) provided reimbursement for dialysis expenses; In low- and middle-income countries, reimbursement of dialysis costs is insufficient to treat all patients with ESRD, and has a disproportionate effect on public health spending.
	Tonelli et al. (2020a)^(24)^	To compare healthcare policies related to transnational dialysis between the US and Canada, focusing on payment, finance, regulation, and organization.	Dialysis care in the US is primarily government-funded and provided predominantly by for-profit private providers; Dialysis care in Canada is also government-funded and primarily provided via public facilities. Differences in health policy areas are associated with considerable variations in clinical outcomes: mortality among patients undergoing dialysis is consistently lower in Canada than that in the US.
	George et al. (2017)^(25)^	To assess the ability to screen for CKD in low- and middle-income countries.	Effective implementation of CKD screening methods remains challenging, requiring further exploration of cost-effectiveness in these countries.
Infrastructure and human resources for renal healthcare	Bello et al. (2017)^(5)^	To assess the current global state of kidney care.	95%, 76%, and 75% of countries provided facilities for HD, PD, and transplantation, respectively; In Africa, 94%, 45%, and 34% of countries offered facilities for HD, PD, and transplantation, respectively; Tests for monitoring CKD in primary care were consistently available in 18% and 8% of countries; The number of nephrologists was variable and low (<10 per million inhabitants) in Africa, Middle East, South Asia, Oceania, and Southeast Asia.
	Hippen et al.(2018)^(26)^	To propose a model for integrating general nephrology practices, transplant centers, and dialysis providers to offer care for patients across the entire spectrum of kidney disease.	The expanded Comprehensive ESRD Care (CEC) model integrates nephrology practices, dialysis providers, and transplant centers, enabling the development of novel strategies to care for all patients with kidney diseases.
	Kaze et al. (2018)^(27)^	To assess the prevalence of CKD in African populations and examine local registration, screening, and care models.	Generalized absence of renal function assessment methods.
	Jardine et al.(2020)^(28)^	To assess the status of CKD care and treatment in South Africa.	Only 2.5 nephrologists per million population (pmp), unevenly distributed with 90% concentrated in three of the nine provinces and 60% in the private sector.
	Lunney et al. (2019)^(29)^	To compare Canada’s ability to provide renal healthcare with that of other countries with similar economic situation.	Most Organization for Economic Co-operation and Development (OECD) countries provide government-funded renal healthcare; Canada has a public fund for treatment and a mixed public-private fund for medications; Healthcare capacity is not homogeneous across provinces.
	Flood et al. (2020)^(30)^	To evaluate healthcare professionals’ perceptions of the quality of renal care provided at a public nephrology center in Guatemala.	The primary challenge is providing high-quality care due to resource constraints; Practitioners reported substantial emotional challenges related to high patient volume and difficult decisions regarding resource allocation.
	Delatorre et al. (2021)^(31)^	To investigate the knowledge and attitudes of primary care physicians regarding the care of patients with CKD in Brazil.	Health consultation and drug treatment were the predominantprevention strategies; 74% of physicians lacked matrix support for CKD.
	Riaz et al. (2021)^(32)^	To assess the global workforce capacity for renal failure care.	The workforce varies based on the country’s income level; The global density of nephrologists is 10.0 pmp; High-income countries have an average of 23.2 nephrologists pmp, whereas low-income countries provide only 0.2 nephrologists pmp.
Public assistance	Bello et al. (2017)^(5)^	To assess the current global status of kidney care.	HD, PD, and transplant services were publicly funded and free in 42%, 51%, and 49% of countries, respectively.
	van der Tol et al. (2019)^(23)^	Compare global government reimbursements for dialysis costs.	The prevalence of patients per million of the population receiving dialysis in low- and middle-income countries increased linearly with Gross Domestic Product (GDP) per capita. However, this prevalence remained substantially lower in these countries when compared to wealthier countries.
	Jardine et al. (2020)^(28)^	To assess CKD care and treatment in South Africa.	Most South Africans (84%) depend on public healthcare; Public health units charge for the service in proportion to income – indigents are exempt from payment.
	Rojahn et al. (2016)^(33)^	To identify public policies for remote monitoring (RM) in the United Kingdom, Germany, Italy, and Spain.	Policies on RM and/or telemedicine addressing non-communicable diseases (NCDs) have been identified and are well-received in all countries surveyed; Pilot projects (primarily on diabetes, chronic obstructive pulmonary disease, and/or heart failure) were established or in planning stages in most countries.
	Santos Junior et al.(2017)^(34)^	To describe the prevalence of patients with ESRD on publicly funded dialysis in Brazil.	From 2008 to 2013, a 25% increase in the absolute number of HD sessions was noted; From 2008 to 2013, the kidney transplant rate increased from 35.2 to 41.6 transplants per year pmp. In 2013, HD was the most frequent therapeutic modality (87.1%), followed by PD (9.2%) and continuous outpatient treatment (3.7%).
	Norouzi et al. (2020)^(35)^	To investigate the effect of including new medications in the reimbursement package for patients with ESRD in US dialysis institutions.	Closure of dialysis facilities was rare during the study period; The impact on patients regarding access to treatment was minimal or almost nil.
	Chuengsaman et al. (2017)^(36)^	To assess key policy development and implementation strategies, such as home PD in Thailand.	The Thai PD First policy saved the lives of nearly 50,000 ESRD patients on dialysis under the universal coverage scheme. Despite ongoing challenges, the program continues to evolve.
	Kanjanabuch et al.(2020)^(37)^	To assess the impact of the PD first policy and reimbursement schemes on dialysis treatment in Thailand.	In 2008, the Thai government launched the PD First policy, providing Thai citizens with universal health coverage for dialysis; After the implementation of the “PD First” policy, the number of patients in dialysis and dialysis centers increased exponentially.

**Table 1B t2:** Papers classified into the main thematic category “Access to renal healthcare and management of chronic kidney disease”

Theme	Authors	Objectives	Main findings
Access to renal health care	Bello et al. (2017)^(5)^	To assess the current state of global kidney care, including policy recommendations for improvement.	95%, 76%, and 75% of countries provided facilities for hemodialysis (HD), peritoneal dialysis (PD), and transplantation, respectively; In Africa, 94%, 45%, and 34% of the countries provided facilities for HD, PD, and transplantation, respectively; Availability of tests for monitoring chronic kidney disease (CKD) in primary care was reported in 18% and 8% of the countries, respectively.
	Rastogi et al. (2021)^(38)^	To evaluate the clinical, quality of life, economic, and social aspects of dialysis modalities for patients with end-stage renal disease (ESRD).	Patients on HD in treatment centers are at an increased risk of arrhythmias, mortality, and hospitalization; Home dialysis can improve patient prognoses. However, eligible patients are generally young and healthy. As home dialysis is not feasible for all patients, public policies in the US advocated its use whenever possible, seeking to improve health outcomes and reduce costs.
	Agudelo-Botero et al. (2020)^(39)^	To describe the access and treatment of patients with ESRD in public hospitals in Mexico and their barriers to reaching dialysis services.	76.9% of patients received HD or PD as their first renal replacement therapy; 64% of the patients on HD received intermittent treatment; Several barriers to accessing treatment were identified, with patients facing economic difficulties.
	Mercado-Martínez et al. (2017)^(40)^	To examine kidney care in Uruguay from the perspective of transplant recipients with CKD and their families.	Those with kidney transplants who are young, reside in the capital, and have private health coverage, consider the care system adequate, efficient, and of good quality; Conversely, retirees or pensioners, those with multiple comorbidities, residing in the countryside, are covered by the public sector, and live in poverty do not consider the care system adequate to their needs; Numerous patients encounter considerable barriers in accessing free, timely, quality care.
	Lin et al. (2017)^(41)^	To analyze associations between PD promotional policies and actual PD selection rates in Taiwan.	The incidence of PD increased from 12.8% in 2006 to 15.1% in 2009, decreasing in 2010 (13.8%) when the hospital accreditation policy was revoked.
	Luyckx et al. (2019)^(42)^	To discuss key strategies to address traditional and non-traditional risk factors of CKD in high-income countries.	In high-income countries, barriers to accessing effective therapies for the treatment of CKD must be eliminated by health policies.
Social, economic, and ethnic inequalities hindering access to renal health care	Crews et al. (2019)^(15)^	To highlight disparities and emphasize the role of public policies and organizational structures in addressing CKD.	Considerable international variation was observed in the distribution of the kidney care workforce; The poorest individuals suffer disproportionately.
	Luyckx et al. (2019)^(42)^	To discuss key strategies for addressing traditional and non-traditional risk factors of CKD and its prevention in high-income countries.	CKD occurs more frequently and progresses rapidly among indigenous, minority, and socioeconomically disadvantaged populations.
	Crews et al. (2020)^(43)^	To highlight inequalities in dialysis care and its consequences in the US; To highlight how public policies can influence inequalities.	Inequalities in access and treatment are pronounced in the US; Racial and ethnic minorities and low-income individuals have limited access to treatment; Recently, new policies have been implemented to minimize inequalities; however, they have not yet reached their full objective.
	Raghavan (2018)^(44)^	To stimulate research and public discussions aimed at creating more humane and appropriate solutions to support undocumented immigrants with kidney failure in the US.	3% of the US population is composed of undocumented immigrants, and 27% of do not have health insurance, with most receiving treatment only in an emergency or life-threatening situation; An urgent global policy is essential to support immigrant health; Given the current world scenario, the chances of reaching a uniform solution in the short or medium term are minimal.
	Huria et al. (2018)^(45)^	To examine inequalities in dialysis-related incidence, treatment, and survival in indigenous communities of New Zealand.	Non-Māori patients undergo temporary dialysis vascular access less frequently than Māori and experience lower mortality rates, even when socioeconomic, demographic, and geographic factors are equivalent.
	Moosa et al. (2021)^(46)^	To discuss the need to recognize the social drivers of noncommunicable diseases in low-income countries, such as inequality and wealth discrimination, and highlight the importance of coordinated multisectoral interventions.	In impoverished communities, the risk of developing CKD begins to increase even before birth and persists throughout life; The burden of diabetes is increasing at a faster rate in lower-middle-income countries compared with high-income countries; Much of this increase in incidence is directly or indirectly related to the consequences of poverty.

**Table 1C t3:** Papers classified into the main thematic category “Strategies for coping with chronic kidney disease and promoting kidney health”

Theme	Authors	Objectives	Main findings
Prevention of CKD	Sola (2017)^(12)^	To evaluate the process of integrating CKD prevention into Uruguay’s National Program for Noncommunicable Diseases.	In 2004, a CKD prevention program was launched. It began with a pilot program at the State Health Services in Montevideo and was under the supervision of an Advisory Committee on Renal Health.
	George et al. (2017)^(25)^	To assess the ability to screen for CKD in low- and middle-income countries.	Low- and middle-income countries are ill-equipped to monitor and manage the consequences of CKD, particularly in its advanced stages; Screening for CKD should be a policy priority in low- and middle-income countries, as early intervention is likely to effectively reduce the high burden of morbidity and mortality associated with CKD.
	Kaze et al. (2018)^(27)^	To assess the prevalence of CKD in African populations and local registration, screening, and care models.	The prevalence of CKD is higher in sub-Saharan Africa than in North Africa. In high-risk populations, the prevalence of CKD is almost twice as high as in the general population; Among the 54 countries evaluated, 32 lacked sufficient data on the population affected by CKD.
	Stel et al. (2017)^(47)^	To examine the correlation between risk factors for CKD and its prevalence on an ecological level, evaluating the reasons for the differences in prevalence among European countries.	Countries with a higher prevalence of CKD also showed higher average scores on risk factors for CKD and vice versa; No association was observed between cardiovascular mortality rates and CKD prevalence.
	Narva (2018)^(48)^	To evaluate the reasons behind the persistently high burden of kidney disease in the US despite extensive clinical guidance, innovative care initiatives, and well-funded awareness campaigns.	The Indian Health Service and the Centers for Disease Control and Prevention reported a 54% reduction in kidney failure among Indian Americans and Alaska Natives with diabetes; Decreased incidence of end-stage renal disease (ESRD) among Indian-Americans was associated with a population-based approach, emphasizing diabetes management within the community and the primary clinical settings.
	Joshi et al. (2017)^(49)^	To highlight that the burden of CKD in low- and middle-income countries is related to system-wide issues that could be reduced by innovative, affordable, and scalable interventions.	A multifaceted approach is necessary, including improvements in socioeconomic determinants of health; Innovative approaches to promoting healthy behaviors, counseling, and education in primary care, alongside using screening technology throughout various processes, are critical.
	Luyckx et al. (2018)^(50)^	To map actions towards the achievement of all United Nations “Sustainable Development Goals,” with the potential to improve the understanding, measurement, prevention, and treatment of renal diseases.	Kidney disease is potentially preventable, and low-cost interventions can minimize adverse outcomes; Diagnosis is often hampered by a lack of awareness among health professionals and people at risk; Universal health coverage, with effective and transparent public policies, is essential to prevent and manage kidney disease.
	Stanifer et al. (2018)^(51)^	To characterize existing models of care for CKD in low-income countries.	National efforts are usually focused on CKD prevention through screening, public awareness campaigns, and education; Among the 12 clinical care models observed, nine targeted individuals with CKD and the remaining three focused on those at risk for CKD; Few rigorous models of CKD care have been reported in low- and middle-income countries.
Planning and future perspectives to address and manage CKD	Maddux (2020)^(52)^	To outline the key factors that may allow the enactment of the 2019 US Presidential Executive Order to evolve appropriately.	Politics should take a broader view of social and systemic factors that affect chronic diseases; Addressing the entirety of the CKD epidemic requires consideration of both internal and external factors within traditional medical-pathophysiological contexts, including the social determinants of health.
	Fukui et al. (2019)^(53)^	To describe the new measures for managing CKD in Japan.	The Japanese “Kidney Disease Control Commission” has established several defined key performance indicators for the healthcare system aimed at reducing the number of new patients with dialysis from 39,000 in 2016 to ≤35,000 in 2028.
	Silva et al. (2020)^(54)^	To investigate the advances and challenges of Brazilian public policies designed to address the progression of CKD and its risk factors.	The field of nephrology requires investments to support the planning of individualized, interdisciplinary, and shared care alongside primary care.
	Wong et al. (2018)^(55)^	To estimate the number of residents with CKD in Singapore by 2035 using a Markov model.	Between 2007 and 2035, the number of residents with CKD is projected to increase from 316,521 to 887,870, with prevalence increasing from 12.2% to 24.3%; It is predicted that patients with CKD stages 1-2 constitute the largest proportion, and undiagnosed cases are expected to decrease from 72.1% to 56.4% owing to improved detection methods.
	Wimalawansa (2019)^(56)^	To estimate the costs of eradicating multifactorial kidney disease (mfKD) and the resulting cost savings, using Sri Lanka as an example.	The annual cost required to eradicate the disease has been estimated at approximately one-tenth of the current operating costs attributed to mfKD; Models focused on preventing chronic diseases can eradicate mfKD within 15 years; Planned measures include basic sanitation, education, poverty and malnutrition alleviation, and regional screening, diagnosis, and intervention programs.
	Wu et al. (2018)^(57)^	To describe Taiwan’s intelligent kidney care system.	Since the inception of the first dialysis, the Taiwan Renal Registry Data System (TRRDS) has facilitated data retrieval to generate knowledge for decision-making; The TRRDS has enabled the development of innovative and successful experiences.
	Venuthurupalli et al. (2018)^(58)^	To examine screening programs and surveillance systems in Australia.	National surveys have shown that the burden of CKD in Australia is a major public health concern; The country currently uses a registry system to monitor patients on renal replacement therapy.
	Tonelli et al. (2020b)^(59)^	To suggest a framework for establishing integrated kidney care programs, focusing on policymakers in low- and middle-income countries.	Principles of the proposed integrated kidney care: Priority should be given to treatments aimed at slowing or preventing the progression of the disease; Treatments for symptom management should be established in parallel with preventive care programs; In lower-middle-income countries, PD should be prioritized over HD owing to its lower cost-benefits.

The majority of papers were published between 2017 and 2020, ranging from eight to ten throughout those years (Figure 2). Only two papers from 2016 and four papers until the end of July 2021 met the selection criteria.

Figure 3 demonstrates that North American policies (excluding Mexico) produced the highest percentage of articles (26%), followed by Asian (19%), Latin and Central American (16%), Africa (5%), Europe (5%), and Oceania (5%). Articles covering multiregional or global aspects of combating CKD accounted for 24% of the total selected papers in this review.

To better address the research question, we categorized the selected articles into three main thematic areas focusing on global approaches to manage CKD: i) structural and financial aspects associated with how renal healthcare is managed at the organizational level; ii) access to renal healthcare and management of chronic kidney disease; and iii) strategies for coping with chronic kidney disease and promoting kidney health. Finally, each category was subdivided into subthemes.

Thematic category 1: Structural and financial aspects associated with how renal healthcare is managed at the organizational level:

- Theme 1: Public expenditure and budget for renal healthcare;
- Theme 2: Infrastructure and human resources for renal healthcare;
- Theme 3: Public assistance.

Thematic category 2: Access to renal healthcare and management of CKD:

- Theme 1: Access to renal healthcare;
- Theme 2: Social, economic, and ethnic inequalities hindering access to renal healthcare.

Thematic category 3: Strategies for coping with CKD and promoting kidney health:

- Theme 1: Prevention of CKD;
- Theme 2: Planning and future perspectives to address and manage CKD.

Tables 1A to C [Table t2] [Table t3]provide detailed descriptions of the selected articles grouped according to the highlighted categories and themes.

## DISCUSSION

### Thematic Category 1: Structural and financial aspects associated with how renal healthcare is managed at the organizational level:

#### Public expenditure and budget for renal healthcare

Public budget allocation models for managing CKD and its complications are strongly associated with the economic situation of each country. Although all surveyed countries provided renal healthcare with government funding,^(23)^ the models for patient allocation and support are different.^(15,29)^

In developed regions with higher incomes, such as the United States (US), Canada, Japan, Australia, and several European countries, the total resources allocated to combat CKD is higher than that in middle- and low-income countries.^(20,21,24,25)^ According to Tonelli et al., dialysis care in the USA and Canada is primarily funded by the government.^(24)^ In the US, services are primarily provided by for-profit private providers, whereas in Canada, they are primarily provided in public health centers.^(24)^

Public investment in kidney health has increased in the low- and middle-income countries. In countries such as Malaysia, the annual public expenditure on end-stage renal disease (ESRD) has increased by 97% within seven years.^(20)^ However, service costs and the number of inpatients served by the health systems in those countries have also markedly increased.^(19)^ Projections indicate that expenditures will increase even further in the near future, posing substantial implications for the financial sustainability of the healthcare system and public health.^(19,20,22)^ However, low- and middle-income countries are addressing this challenge using different and often inefficient approaches.^(59)^

#### Infrastructure and human resources for renal healthcare

In general, even among countries in similar stages of economic development, or among states or provinces of the same country, important variations exist in workforce and infrastructure allocation models. These variations are intricately dependent on the internal policies of each nation.^(26,29)^

In low- and middle-income regions, a shortage of health professionals and inadequate treatment facilities and methods for renal function assessment and screening exist.^(27,28)^ Bello et al., using data from 125 countries, representing approximately 93% (6.8 billion) of the world’s population, highlight the substantial interregional and intraregional variability in the current capacity for renal care worldwide.^(5)^ Although the world averages indicate that 95%, 76%, and 75% of the countries provide facilities for hemodialysis (HD), peritoneal dialysis (PD), and transplantation, respectively, in African countries, only 45% and 34% of them had facilities for PD and transplantation, respectively. Regarding primary healthcare, crucial for monitoring and controlling kidney diseases, only 18% of the studied countries have full-time tests for estimating glomerular filtration rate, and less than 8% offer tests for monitoring proteinuria.^(5)^

The number of nephrologists is also a concern worldwide.^(14)^ Riaz et al. reported a mean global density of 10.0 nephrologists per million population (pmp). In high-income countries, the average was 23.2 nephrologists pmp, whereas in low-income countries, the average was only 0.2 pmp.^(32)^

The scarcity of renal health professionals is often accompanied by severe infrastructure limitations and poor access to medication.^(28,32)^ This challenging reality affects the physical and mental health of medical and nursing teams, and also subjects them to various dilemmas such as burnout and moral distress, as highlighted by Flood et al.^(30)^

Another relevant aspect is training and updating primary healthcare professionals to effectively contribute to the prevention of kidney diseases. For example, research conducted by Delatorre et al. in eight Brazilian cities revealed that less than 60% of physicians recognized smoking and obesity as risk factors for CKD.^(31)^

#### Public assistance

Kidney disease is highly prevalent, affects the entire lifespan, and has substantial financial implications for patients. Most individuals worldwide depend on government support for treatment across all regions.^(28,33)^ In all countries, particularly in middle- and low-income areas, free and universal health coverage is critical and often the sole means by which patients with kidney disease patients can receive treatment.^(50)^ However, according to Bello et al., among 125 countries representing 93% of the world’s population, only 42% provide publicly funded HD services, 51% provide PD, and 49% provide transplantation services.^(5)^ In a similar study involving 90 countries, van der Tol et al. found that although not all countries provide free services, approximately 90% provide some form of reimbursement for patients undergoing dialysis. However, the authors cautioned that in low- and middle-income countries, reimbursement for dialysis expenses is insufficient for all patients with ESRD.^(23)^

Current models of economic support for patients with renal disease vary widely globally. In Brazil, patients with CKD have free access to all treatment phases.^(31,34)^ Although 84% of the population in South Africa depends on public healthcare, the financing model is such that public health units charge for the service in proportion to the patient’s income.^(28)^ Other countries have sought alternatives to reduce costs and increase the number of patients requiring dialysis. For example, the “Peritoneal Dialysis First” (DP First) policy launched by the Thai government in 2008 showed notable outcomes, making it possible to provide universal health coverage for dialysis to almost all patients in need.^(36)^

Public healthcare models differ considerably in wealthier countries, such as the USA and Canada. Although Canada does not provide universal healthcare, the government offers a public fund for CKD treatment and a mixed public-private fund for medications. In the US, most individuals depend on personal or employer-provided health insurance.^(29,35,38)^ Both countries have pursued policies to expand access to healthcare for patients with kidney disease. For example, in the US, Medicare and Medicaid systems have recently begun to include several essential medications in the reimbursement package for patients with kidney disease.^(29,35)^

## Thematic category 2: Access to renal health care and management of CKD:

### Access to renal healthcare

Globally, a shortage of methods exists for assessing renal function, especially evident in less affluent countries, where accessing quality care faces economic and political limitations.^(27,38)^ Moreover, major inequalities persist in access to and treatment of kidney diseases in almost all countries.^(41,42)^

Adequate disease registration and population mapping are crucial for developing effective healthcare policies regarding NCDs such as CKD.^(41,50)^ Although wealthier countries manage this well, numerous poorer countries, notably in Africa, lack proper facilities for disease registration, screening, and care.^(27)^

In developing countries, access to renal healthcare has improved in recent years; however, the progress remains slow. A study in Uruguay by Mercado-Martínez et al. revealed disparities in renal health services. Although those with higher incomes and urban residents find access and quality satisfactory, older individuals in rural areas, relying on public health services perceive access and quality as unsatisfactory. The authors reported that despite the improvements observed over the years, a large percentage of the renal population faces barriers to accessing free and quality care.^(40)^ In Brazil, a similar situation has been observed, with an increase in the number of HD sessions and kidney transplant rates. Furthermore, considerable differences have been noted among Brazilian states and local regions, where access to health is usually more difficult for populations residing in the northern and northeastern regions of Brazil.^(9,34)^ Agudelo-Botero et al. reported that in Mexico, individuals with greater economic difficulties encounter substantial barriers to accessing renal healthcare. They urged governments to implement specific public policies for CKD, primarily aimed at improving access and preventing/minimizing complications.^(39)^

Notably, contrary to expectations, substantial barriers to accessing effective therapies have also been observed in higher-income countries, despite considerable investments in the treatment of kidney diseases.^(53)^ For example, although treatment costs are partially covered by the public sector in several countries, especially in urgent and emergency cases, most patients depend on health insurance to fund their treatment.^(5,33,47)^

### Social, economic, and ethnic inequalities hindering access to renal healthcare

According to Luyckx et al., CKD tends to occur more frequently and progresses rapidly among indigenous, minority, and socioeconomically disadvantaged populations.^(42)^ Crews et al., in their 2019 study, confirm this observation, finding that individuals with CKD from disadvantaged backgrounds suffer disproportionately compared to those with greater purchasing power, regardless of whether the country has a universal public health system.^(15)^

A study in New Zealand reported that Māori patients underwent treatment with vascular access for temporary dialysis more frequently than non-Māori patients. This study showed that Māori patients have a higher mortality rate than non-Māori patients, even when socioeconomic, demographic, and geographic factors are equivalent. This highlights the need to investigate other important factors such as social, genetic, lifestyle, and ethnic considerations.^(45)^ In the US, Crews et al. demonstrated that racial and ethnic minorities, and minorities with lower purchasing power, have less access to CKD treatment.^(43)^

Raghavan highlighted the inadequate health support for immigrants, refugees, and others who left their home countries for humanitarian reasons,^(44)^ particularly in the USA, where approximately 3% of the population consists of undocumented immigrants. Among them, 25% do not have health insurance and receive treatment only during emergencies and life-threatening situations.^(44)^ It is essential to highlight that such issues are rarely addressed in health studies, especially kidney diseases. With recent migration patterns from impoverished or conflict-ridden regions to wealthier areas,^(11)^ urgent discussions among global organizations such as the UN and WHO are necessary to develop policies and strategies to mitigate imminent health crises.

Although some countries have recently implemented policies to minimize inequalities, they remain few, limited in scope, and have not yet reached their main objectives.^(44,46,52)^ Strong political will is crucial for addressing profound social, economic, and ethnic inequalities in almost all countries.^(15,43,46,49)^

A consensus among researchers is the need for urgent and greater engagement of the scientific community and social and political organizations to promote new health policies aimed at achieving more equitable and humane access to treatment worldwide.^(44,50,54)^ Therefore, it is vital to recognize the social and economic factors that lead to NCDs, especially in low-income countries, such as inequality, poor wealth distribution, and access to quality education.^(52)^ It is crucial that all politicians and decision-makers begin to perceive these issues through a broader and multifaceted prism such that proper models, projects, and fundamental actions can be developed and implemented to prevent and control diseases such as CKD.^(46,49)^

## Thematic category 3: Strategies for coping with CKD and promoting kidney health:

### Prevention of CKD

The studies listed in table 1C show that few countries have implemented adequate measures to prevent CKD.^(12,51)^ In general, researchers agree that much is to be done concerning public policies that explicitly focus on this topic.^( 27,39,34,50)^

Research conducted across various regions worldwide highlights the critical need for implementing public policies focused on CKD, primarily emphasizing prevention, minimizing complications, and supporting patients. This urgency is recognized irrespective of the economic conditions of the countries involved. Narva stated that despite financial investments, extensive clinical guidance, and efforts to improve care and raise public awareness regarding CKD in the US, little progress exists in alleviating the burden of kidney disease. This suggests the need for an in-depth review of current policies.^(48)^ One of the recent milestones in the USA seeking to improve public policies associated with the prevention and treatment of CKD was the enactment of the 2019 Presidential Executive Order “Advancing American Kidney Health.” The Executive Order was characterized by a set of initiatives to reduce the incidence of CKD, increase dialysis options, and encourage kidney transplantation programs. According to Crews et al., this initiative has already resulted in gains for health professionals, institutions, and patients, and has the potential to profoundly transform the current scope of treatment and clinical practice in nephrology.^(43)^ The study conducted by Stel et al. highlights that despite notable social progress, European countries still lack more efficient, equitable, and comprehensive public policies regarding CKD. The authors advocate for a stronger approach emphasizing the management of risk factors to prevent CKD and mitigate progression in different countries of the continent.^(47)^

The most critical situation is perceived in the poorest regions and countries of the world, where public policies tend to be inefficient and sometimes nonexistent. Joshi et al. observed a general absence of strong political will in numerous countries, hindering the development of efficient solutions for preventing and managing CKD.^(49)^ Other research aimed at low- and middle-income countries have shown similar scenarios.^(14,39)^ When evaluating existing healthcare models in low-income countries, Stanifer et al. observed that although most countries have strategies for managing CKD, the models implemented for the prevention and care exhibit deficiencies in several aspects. These include the need for improvements in primary healthcare, inpatient follow-up protocols, and the implementation of national awareness-raising policies to address the population.^(51)^ George et al. showed that screening for CKD should be a political priority in low- and middle-income countries because early intervention can notably reduce the high economic and social burden associated with CKD morbidity and mortality.^(25)^ Similar conclusions were reached by Santos Junior et al. and Ismail et al., who assessed the prevalence and magnitude of the economic burden related to kidney disease treatment in Brazil and South Asian countries, respectively.^(20,22,34)^

### Planning and future perspectives to address and manage CKD

Although numerous studies highlight the necessity for improved policies with systematic approaches to combat CKD, especially in low-income countries,^(25,27,28,31,34,40,42,46,48-50,52,54)^ there is also a gradual increase in the importance of the issue among policymakers. Consequently, several countries are seeking innovative solutions that can be incorporated into future protocols and policies for the prevention and treatment of CKD.

For example, in the US, an important milestone in the care policy for patients with CKD was reached with the 2019 Presidential Executive Order. This initiative entails a meticulous plan aimed at reducing end-stage renal disease in the country in the coming years.^(52)^

Australia also serves as a model for systematic policy planning, focusing on the medium- and long-term goals. Venuthurupalli et al. demonstrated the considerable progress of Brazil in effectively managing CKD, transitioning from a basic screening and patient follow-up model to sustainable and efficient long-term surveillance.^(58)^

The Taiwan Renal Registry Data System , established since the country’s first, is a dynamic learning model capable of collecting, accumulating, analyzing data, and intelligently interpreting results. This innovation in health system management enables the bodies responsible for the implementation and modernization of healthcare policies enables proactive planning and adjustment of local and national protocols, enhancing clinical outcomes and cost-effectiveness of kidney disease treatment.^(57)^

Japan provides another notable example of concrete actions for future planning and combating. Since 2018, the government established a series of performance indicators to be achieved by the health system to reduce the number of new patients undergoing dialysis from 39,000 in 2016 to under 35,000 by 2028. To achieve these goals, a special commission outlined core actions for increasing public awareness, enhancing regional health provisions, improving medical care, developing human resources, and promoting research and development of new techniques and treatments.^(53)^

Of the various studies described in table 1C, three stand out for their substantial contributions. They propose scientific models for studying CKD and its progression over the following decades and provide cost estimates for managing the disease. These elements are crucial for planning future public actions and policies.

In 2018, Wong et al. published a study aiming to estimate the prevalence of CKD in Singapore by 2035. They proposed a mathematical model based on the Markov Model to simulate various scenarios regarding prevalence, incidence, mortality, transition between disease stages, and disease detection (screening) rates. The model projects an increase in the number of residents from 316,521 to 887,870, and prevalence from 12.2% to 24.3% from 2007 to 2035.^(55)^ These projections are substantial for a country with approximately 6 million people today.

In 2019, Wimalawansa proposed a model to estimate the costs of eradicating multifactorial kidney disease (mfKD) and the resulting savings from efficient actions. Using the evolution of mfKD in Sri Lanka as a case study, the authors demonstrated that the annual cost required to eradicate the disease would be approximately one-tenth of the current operating cost, owing to these conditions.^(56)^

Finally, in 2019, a group of 16 researchers from different regions of the world proposed a model for establishing integrated renal care programs, focusing on the demands and needs of policymakers in low- and middle-income countries. The model is based on the principle of integrated kidney care, in which i) treatments to delay or prevent the progression of kidney disease should have priority, ii) treatments to control symptoms should be established alongside preventive care programs, and iii) for lower-middle-income countries, PD should be prioritized over HD owing to its cost-benefit ratio. Adherence to this model can provide health policy managers with a tool to describe and justify the principles underlying the establishment of a national renal care program.^(59)^

## FINAL CONSIDERATIONS

This integrative review presents an overview of several key issues in the fight against CKD.

The prevention and treatment of CKD involve numerous actors and actions in various complex scenarios. The analysis of selected papers within the scope of the chosen thematic categories showed that despite the efforts of several countries, kidney diseases have been neglected in the world political agenda, highlighting the need to increase awareness among governments and the general population.

Strategies and policies for managing CKD vary widely among. CKD management models are closely linked to the economic situation of each nation or region. Owing to the high cost of managing the disease, low-income countries and areas tend to have insufficient infrastructure, healthcare professionals per million inhabitants, and treatment facilities and methods for assessing kidney function and patient triage. Expanding efforts in primary healthcare is suggested to minimize CKD treatment costs. Paradoxically, only a small percentage of the world’s poorest countries provide adequate primary care.

High treatment costs imply the dependence on public funding for most patients with CKD. Thus, free and universal health coverage is essential for accessing proper treatment, especially for the neediest patients with renal disease. Unfavorable socioeconomic contexts worldwide are often combined with the absence of policies aimed at improving the population health conditions, exacerbating the situation.

Recently, most developing countries have made considerable efforts to expand access to renal healthcare. However, a large proportion of the renal population encounters considerable barriers to accessing free and quality care in numerous countries. Even in the highest-income countries, patients have difficulty accessing treatment, with a considerable portion of the population depending on health insurance.

Social, economic, and ethnic inequalities strongly correlated with the occurrence of CKD in many regions of the world. Minority groups, indigenous populations, immigrants, refugees, and other socioeconomically disadvantaged groups often suffer the most from a lack of general health support, particularly for CKD treatment.

Overall, most authors agree that the development of new and better health policies with adequate planning to manage CKD is crucial. Such policies must be based on systematic approaches, particularly for low-income countries. Nevertheless, most nations have adopted a reactive approach to the evolution of the disease, which is insufficient and could have substantial health and economic consequences, especially considering the projections of a sharp increase in the prevalence of CKD worldwide in the near future.

Global political agents must recognize the importance of the detrimental effects of inequality, wealth distribution disparities, and limited access to education on population health. To do so, the scientific community alongside social and political organizations, should advocate for new health policies to ensure equitable access to and treatment of CKD, fostering a global effort or effectiveness and fairness.
